# Hydrogel-forming microneedles enhance transdermal delivery of metformin hydrochloride

**DOI:** 10.1016/j.jconrel.2018.07.009

**Published:** 2018-09-10

**Authors:** Eman M. Migdadi, Aaron J. Courtenay, Ismaiel A. Tekko, Maelíosa T.C. McCrudden, Mary-Carmel Kearney, Emma McAlister, Helen O. McCarthy, Ryan F. Donnelly

**Affiliations:** a School of Pharmacy, Queen's University Belfast, 97 Lisburn Road, Belfast BT9 7BL, UK; b School of Pharmacy, Applied Science Private University, Amman, Jordan; c School of Pharmacy, University of Aleppo, Aleppo, Syria

**Keywords:** Metformin HCl, Hydrogel-forming microneedles, Transdermal delivery

## Abstract

We investigated, for the first time, the potential for a hydrogel-forming microneedle (MN) patch to deliver the high-dose drug metformin HCl transdermally in a sustained manner. This may minimize some gastrointestinal side effects and small intestine absorption variations associated with oral delivery. Patches (two layers) were assembled from a lyophilised drug reservoir layer, with the MN layer made from aqueous blend of 20% w/w poly (methylvinylether-*co*-maleic acid) crosslinked by esterification with 7.5% w/w poly (ethylene glycol) 10,000 Da. >90% of metformin was recovered from homogeneous drug reservoirs. Drug reservoir dissolution time in PBS (pH 7.4) was <10 min. MN penetrated a validated skin model Parafilm® M consistently. Permeation of metformin HCl across dermatomed neonatal porcine skin *in vitro* was enhanced by using MN. The combined MN and metformin HCl reservoir patch (containing 75 mg or 50 mg metformin HCl, respectively) delivered 9.71 ± 2.22 mg and 10.04 ± 1.92 mg at 6 h, respectively, and 28.15 ± 2.37 mg and 23.25 ± 3.58 mg at 24 h, respectively.In comparison, 0.34 ± 0.39 mg and 0.85 ± 0.68 mg was delivered at 6 h, respectively, and 0.39 ± 0.39 mg and 1.01 ± 0.84 mg was delivered at 24 h, respectively, from a control set-up employing only the drug reservoirs. *In vivo*, metformin HCl was detected in rat plasma at 1 h post MN application at a concentration of 0.62 ± 0.51 μg/mL, increasing to 3.76 ± 2.58 μg/ml at 3 h. A maximal concentration of 3.77 ± 2.09 μg/ml was achieved at 24 h. C_ss_ was 3.2 μg/mL. Metformin transdermal bioavailability using MNs was estimated as 30%.Hydrogel-forming MN are a promising technology that has demonstrated successful transdermal delivery of metformin HCl. Potential clearly exists for administration of other high-dose drugs using this system.

## Graphical abstract

**Figure:**
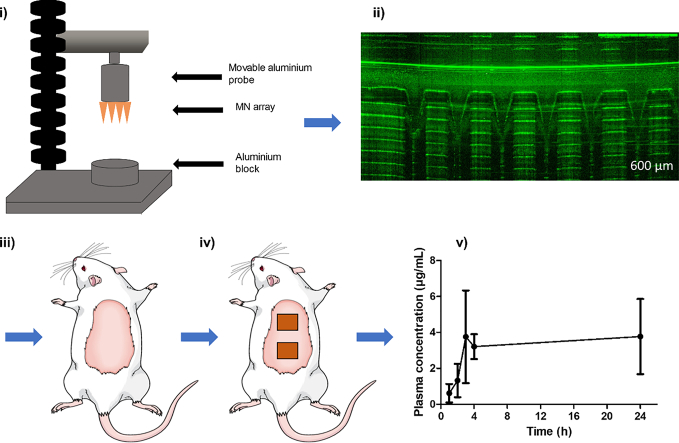

## 1 Introduction

Hydrogel-forming microneedle arrays (MNs) are typically fabricated from aqueous blends of poly (methylvinylether/maleic acid) and poly (ethylene glycol) *via* a micromoulding process using silicone moulds that have been prepared by laser engineering technology [1,2]. Hydrogel-forming MNs, contain no drug themselves, but swell in skin to allow diffusion of drug contained in an attached reservoir layer to the dermal microcirculation for systemic absorption [1]. Many studies have demonstrated the ability of hydrogel-forming MNs to enhance transdermal delivery of a variety of molecules, such as small hydrophilic drugs, including caffeine, theophylline, methylene blue and metronidazole [1]. In addition, high molecular weight substances, such as insulin and bovine serum albumin [1] and high-dose drugs, like ibuprofen [3] and, recently, donepezil have also been delivered [4].

Hydrogel-forming MNs, once applied to the skin, can be withdrawn intact, leaving no polymeric residues behind. This represents a considerable advantage in comparison to dissolving MNs [5]. Hydrogel-forming MNs do not become blocked by compressed dermal tissue upon application, in comparison to hollow MNs [1]. Hydrogel-forming MNs could overcome some of the limitations typically associated with coated MNs, such as extremely reduced MN loading capacity, difficulty in achieving accurate drug coating and controlling rate and extent of drug release [1]. This technology offers a simplified one-step application process, in comparison to uncoated solid MNs that require a two-step application [1].

The use of hydrogel-forming MNs is not restricted to drug delivery. The capability of these MNs to imbibe skin interstitial fluid (ISF) implies that these MNs could be used to extract drug molecules of interest from the skin for subsequent analysis. Drug concentrations in ISF often reflect those in plasma [6], so this technology could prove of great use in blood-free patient drug monitoring and may overcome many limitations associated with direct blood sampling. This is expected to be advantageous for specific patients, such as neonates and the elderly [5]. Indeed, hydrogel-forming MN arrays have been used for successful extraction and quantification of drug substances, such as theophylline, caffeine, glucose and lithium from skin *in vitro* and *in vivo* [7,8].

Metformin HCl [1, 1-dimethyl biguanide hydrochloride] is the most widely prescribed drug for treatment of individuals with type II diabetes mellitus. It is recommended, in combination with lifestyle modification (diet, weight control and physical activity), as a first line oral therapy [9,10,21]. Metformin HCl acts by minimizing insulin resistance, particularly in the liver and in skeletal muscle. It inhibits hepatic gluconeogenesis, increases peripheral insulin sensitivity in insulin-sensitive tissues, such as adipose tissue and muscle and enhances peripheral glucose utilisation [[11], [12], [13], [14]].The most severe side effect of metformin relates to its association with lactic acidosis, particularly in patients with renal and cardiac impairment. Metformin HCl can also cause significant gastrointestinal side effects, including vomiting, diarrhoea, abdominal pain, drowsiness, stomach pain, flatulence and loss of appetite [15].

Recent evidence has indicated that the gastrointestinal tract is an important site of action of metformin HCl. Metformin increases glucose uptake and utilisation in the human intestine, resulting in an increase in lactate production in enterocytes [16,17]. However, the gastrointestinal tract is also the site of an important adverse reaction to metformin, which is the intolerance that often limits metformin dosing or use completely. Metformin intolerance may relate to different mechanisms, including altered transport of serotonin or histamine, local metformin accumulation in enterocytes, increased bile acid exposure in the colon and altered gut microbiome [18,19]. The reduction in bile acid absorption has been suggested as a mechanism through which chronic metformin treatment can lower cholesterol levels [20,21]. It has also been suggested that an increased luminal bile salt concentration would have an osmotic effect, which could lead to the diarrhoea associated with metformin treatment [20].

Metformin HCl is a low potency, high-dose drug [22]. It can be prescribed as 500 mg, 850 mg and 1000 mg tablets. In adults, it is often started at the 500-mg dose and increased weekly until the maximum tolerated dose is achieved, normally 2 g/day, depending upon patient response.It has a reported oral bioavailability of 50–60% under fasting conditions [23]. However, metformin HCl is generally recommended to be taken with meals, as it decreases glucose absorbance. Notably, though, food decreases the absorption of metformin HCl in the small intestine [23], which can result in variations in absorption profile and, in turn, glucose control.

This study investigates the ability of hydrogel-forming MNs to deliver metformin HCl transdermally *in vitro* and *in vivo*. Hydrogel-forming MNs may allow transdermal delivery of metformin HCl in a sustained fashion, which may in turn help minimize some gastrointestinal side effects and avoid the small intestine absorption variations associated with the oral delivery route.

## 2 Materials and methods

### 2.1 Materials

Gantrez® S-97, which is poly (methylvinylether/maleic acid) (PMVE/MA) with molecular mass of 1,500,000 Da was a gift from Ashland, Kidderminster, UK.Poly (ethylene glycol) (PEG), molecular weight 10,000 Da, was purchased from Sigma-Aldrich, Steinheim, Germany. Sodium carbonate (Na_2_CO_3)_ was purchased from BDH Laboratory Supplies, London, UK. HPLC grade methanol, acetonitrile and triethylamine were purchased from Sigma Aldrich, Dorset, UK. Metformin HCl was purchased from Tokyo Chemical Industry Co., LTD, Tokyo, Japan. Cryogel SG3 gelatin was purchased from PB Gelatins, Pontypridd, UK. Pearlitol® 50C-Mannitol was purchased from Roquette, Lestrem, France. Depilatory cream (Boots Expert®) was purchased from The Boots Company PLC, Nottingham, UK. Parafilm® M was purchased from Bemis, Neenah, USA. All other chemicals used were of analytical reagent grade.

### 2.2 Fabrication of hydrogel-forming microneedles (MNs)

An aqueous stock solution of 40% w/w of Gantrez® S-97 (PMVE/MA) was prepared and used to produce a blend containing 20% w/w of Gantrez® S-97, 7.5% w/w of PEG 10,000 and 3% w/w of Na_2_CO_3_ in deionized water. This gel (0.5 g) was carefully poured into pre-formed silicone moulds (11 × 11 needle array density, 600 μm height, 300 μm width at the base and 150 μm interspacing) and the aluminum lid screwed on, before being centrifuged for 15 min at 3500 rpm. After centrifugation, the nascent MNs were dried at room temperature for 48 h. The micromoulds containing the formed MN were then heated at 80 °C for 24 h to induce ester-based crosslinking between PEG and PMVE/MA. Upon cooling, the silicone moulds containing the PEG-PMVE/MA MN arrays were removed from the aluminum holder and the silicone moulds were then peeled away. The sidewalls formed by the moulding process were removed using a heated blade, as described previously [1,3,24].

### 2.3 Mechanical strength of MNs

**Fig. 1:**
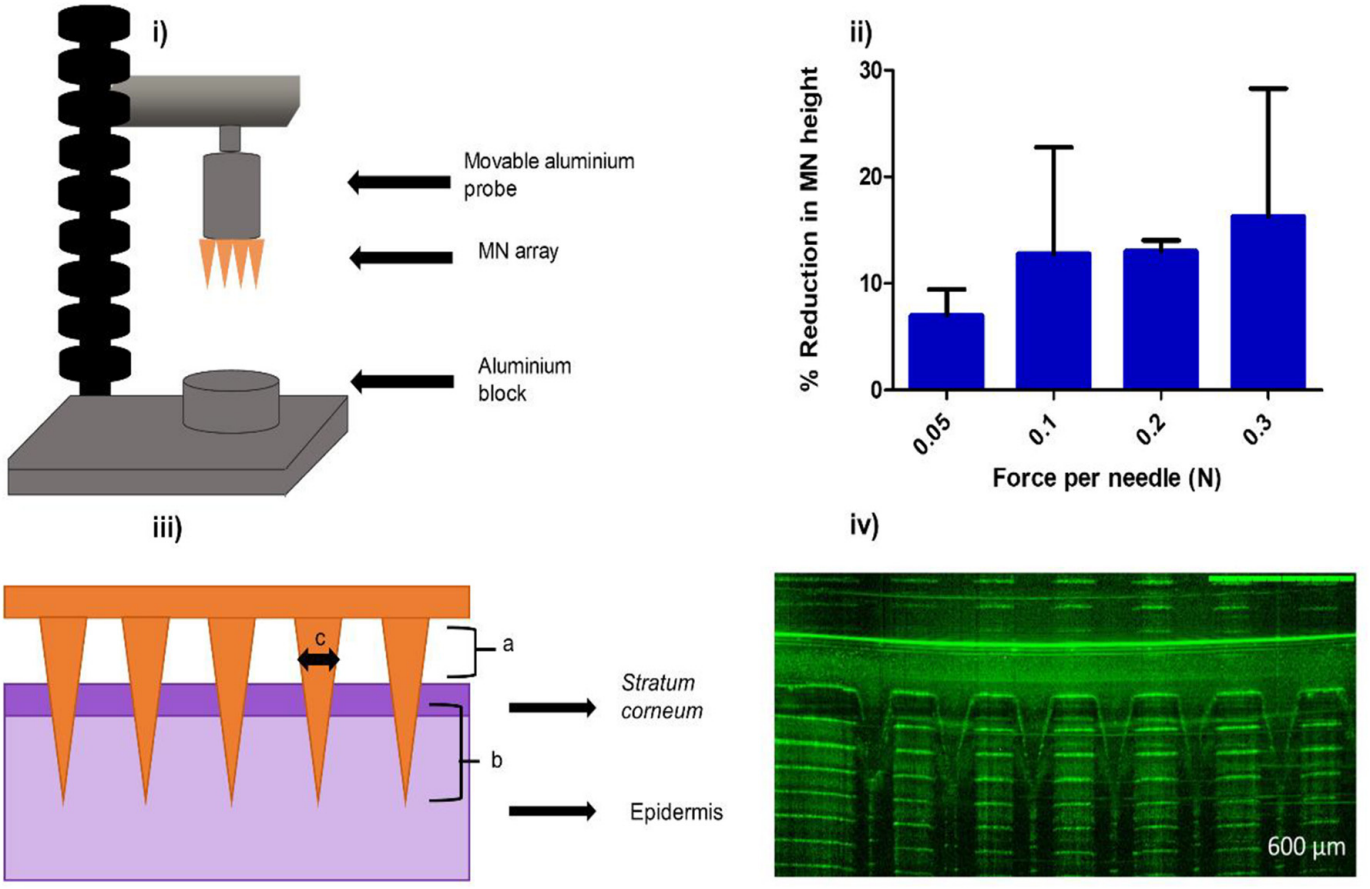
(i) Measuring the mechanical strength of MNs using the Texture Analyser (TA). (ii) Representative bar chart of percentage reduction in the height of needles of MNs measured following the application of different forces using the Texture Analyser (means ± SD, *n* = 3). (iii) Diagrammatic representation of the measurements that are possible from the optical coherence tomographic images of MN penetration into skin, namely; a, the distance between the lower MN base plate and the *stratum corneum* (*SC*); b, the depth of MN penetration into the skin; and c, the width of the micropore created.(iv) The insertion of MNs array in Parafilm® M using OCT.

An axial compression load was applied to the MN arrays (11 × 11) using a TA. XT-plus Texture Analyser (TA, Stable Micro System, Surrey, UK), (Fig. 1 (i)). MN arrays were attached to the moving testing probe of the TA using double-sided adhesive tape (Henkel Ltd., Cheshire, UK). The test station pressed MN arrays against a flat block of aluminum with different compression forces of 0.05, 0.1, 0.2 and 0.3 N/needle for 30 s at a rate of 0.5 mm/s. Pre-test and post-test speeds were 1.0 mm/s and the trigger force was 0.049 N. All MNs of each array were visually examined using the digital microscope (Leica EZ4W, Leica, Wetzlar, Germany). The height of the MNs before and after testing were measured using the software of the digital microscope and the percentage change in the MN height was calculated [[1], [2], [3],^24^].

### 2.4 Skin insertion study using optical coherence tomography

The penetration properties of MN arrays into a validated skin model, Parafilm® M [25], were studied using an optical coherence tomography (OCT) microscope (Michelson Diagnostics Ltd., Kent, UK). A sheet of Parafilm® M was folded to yield an eight-layer film (1.0 mm thickness), placed on a sheet of poly(ethylene) (for support) and covered with aluminum foil. The MN arrays were placed this assembled film of Parafilm® M and fixed in place using adhesive tape. The insertion of MN arrays was carried out manually using thumb pressure for an application period of 30 s. The inserted MNs were immediately visualised using the OCT microscope. The swept-source Fourier domain OCT system has a laser center wavelength of 1305.0 ± 15.0 mm, facilitating real-time high-resolution imaging. The images were analysed using the imaging software ImageJ® (National Institutes of Health, Bethesda, USA). The scale of the image files obtained was 1.0 pixel = 4.2 μm. OCT is employed to allow accurate measurements of the distance between the lower MN base plate and the skin's *stratum corneum* (*SC*) (a), the depth of MN penetration (b) and the width of pore created (c) (Fig. 1 (iii)). The insertion of 12 individual MNs was measured [[1], [2], [3],^24^,^25^].

### 2.5 Fabrication of lyophilised metformin HCl reservoirs

Metformin HCl-loaded drug reservoirs were fabricated using varying concentrations of metformin HCl, gelatin, mannitol and deionized water (Table 1). The components were hand-mixed and then sonicated at 37 °C for 60 min. The resulting formulations were cast (300 mg, 150 mg or 100 mg) into open-ended cylindrical moulds with a diameter of 8 mm and depth 4 mm, then frozen at −80 °C for a minimum of 60 min. Formulations were then lyophilised in the freeze drier (Virtis™ Advantage XL-70 bench top lyophiliser, SP Scientific®, Pennsylvania, USA), according to the following regime: Primary drying for 90 min at a shelf temperature of−40 °C, drying for 90 min at a shelf temperature of −30 °C, drying for 90 min at a shelf temperature of −20 °C, drying for 530 min at a shelf temperature of −10 °C, drying for 90 min at a shelf temperature of 0–10 °C. Secondary drying was carried out for 660 min at a shelf temperature of 25 °C and a vacuum pressure of 50 mTorr [21].

**Table 1:** Formulations of metformin HCl-containing drug reservoirs.

Formula (F) number	Metformin HCl (%w/w)	Gelatin (%w/w)	Mannitol (%w/w)	Water (%w/w)	Formula weight (mg)
F1	40	10	13	47	300
F2	40	10	13	47	150
F3	45	7	3	45	300
F4	45	7	3	45	150
F5	50	5	3	42	300
F6	50	5	3	42	150
F7	35	10	3	52	300
F8	35	10	3	52	150
F9	30	15	3	52	300
F10	30	15	3	52	150
F11	55	5	2	38	300
F12	55	5	2	38	150
F13	50	5	3	42	100

### 2.6 Characterisation of lyophilised metformin HCl reservoirs

Lyophilised metformin HCl reservoirs were characterised visually using the digital microscope. The reservoir thickness was measured using the software of the digital microscope. The time to complete dissolution of the reservoir in PBS (pH 7.4) was determined visually by placing one reservoir of each formula in 20 ml of PBS (pH 7.4) and recording the dissolution time.

### 2.7 The effect of lyophilisation on metformin HCl stability

Lyophilised reservoirs, three of formula F6 (containing 75 mg metformin HCl) and three of formula F13 (containing 50 mg metformin HCl) were placed in individual vials containing 20 ml of PBS (pH 7.4) and stirred using a magnetic stirrer at 200 rpm. After complete dissolution, samples were taken, filtered through 0.2 μm filters and appropriately diluted. Samples were analysed by the validated HPLC method detailed below and the percentage recovery of metformin HCl was calculated.

### 2.8 *In vitro* permeation of metformin HCl

Franz diffusion cells were used to assess permeation of metformin HCl from lyophilised reservoirs through hydrogel-forming MNs and across dermatomed neonatal porcine skin. Skin samples were obtained from stillborn piglets and immediately (<24 h after birth) excised, trimmed to the desired thickness (approximately 350 μm) using an electric dermatome (Integra Life Sciences™, Padgett Instruments, NJ, USA) and frozen at −20 °C until use. MN arrays, (11 × 11 needle array density, 600 μm height, 300 μm width at the base and 150 μm interspacing), were inserted using manual pressure for 30 s. PBS (pH 7.4), 20 μL was then placed on the top of the array to promote adhesion of the lyophilised reservoir, containing either 75 mg (F6) or 50 mg (F13) of metformin HCl, to the MN baseplate. A cylindrical stainless steel weight (11.0 g) was placed on top of the MN array to prevent MN expulsion and the donor compartment of the apparatus was then clamped onto the receiver compartment. The donor compartment and sampling arm were sealed using Parafilm® M. The receiver compartment contained 12.1 ml of PBS (pH 7.4), degassed prior to use and pre-heated to 37 °C ± 1 °C. This ensures complete contact between receiver fluid and porcine skin. Syringes (1.0 mL) with 8.0 cm needles were used to remove 200 μL of the Franz cell contents at pre-determined time intervals and pre-warmed PBS (pH 7.4) was subsequently added to replace this. Control Franz cells were assembled in the same manner, but without the application of MN arrays. Instead, the drug-loaded reservoir was applied directly to the dermatomed porcine skin. All samples were centrifuged at 14,000 rpm using an Eppendorf MiniSpin® centrifuge (Eppendorf UK Limited, Stevenage, UK) for 10 min and diluted prior to HPLC analysis [3,4].

### 2.9 *In vivo* study

Female Sprague-Dawley rats weighing 230.67 ± 17.05 g were acclimatized for 7 days prior to the *in vivo* study. Animals were separated into two groups (*n* = 8 per group). In the oral control group, animals received an oral solution of metformin HCl (100 mg/kg) based on individual rat weight, [26]. The second group was the MNs transdermal treatment group, in which rats were treated with two hydrogel-forming MN patches. To minimize interference of rat hair with MN application in the second group, it was removed from the back region 4 h prior to the experiment. Firstly, electric hair clippers were used to remove the bulk hair, then depilatory cream was applied to remove any residual hair that may otherwise interfere with MNs insertion [1,27]. To facilitate MN application, rats were sedated using gas anesthesia (2–4% (v/v) isoflurane in oxygen). Rat skin was pinched and two MN arrays, mounted on adhesive foam borders, were inserted into the back of each rat using firm finger pressure. Metformin HCl-loaded reservoirs with a drug loading of 50 mg were applied to the back of the arrays covering the area exposed to the MNs. To secure the integrated patch in place, an occlusive dressing layer (Tegaderm™, 3 M, St Paul, Minnesota, USA), was placed on top of the MNs and Micropore™ tape (3 M UK Plc, Bracknell, Berkshire, UK) was used to wrap the back of the animals. All animals in the two groups were fasted for the 24 h experiment duration. Blood samples were taken *via* tail vein bleeds at pre-defined time intervals: 1, 2, 3, 4 and 24 h with a maximum of 200 μL collected at each sampling point into heparinized tubes. The MN arrays were kept in place for 24 h. In accordance with the Project Licence, a staggered study design was employed, with 8 animals per treatment group and a maximum of *n* = 3 blood samples for each rat. Rats were bled at maximum twice daily.Blood samples were taken from the first four rats at 1 h and 3 h. The other four animals were bled at 2 h and 4 h and all animals (*n* = 8) were sampled at 24 h. These samples were processed as detailed below, prior to HPLC analysis. Approval for animal experiments was obtained from the Committee of the Biological Services Unit, Queen's University Belfast. The work was carried out under Project Licence PPL 2794 and Personal Licence PIL 1466. All *in vivo* experiments were conducted according to the policy of the Federation of European Laboratory Animal Science Associations and the European Convention for the protection of vertebrate animals used for experimental and other scientific purposes, with implementation of the principles of the 3Rs (replacement, reduction and refinement).

### 2.10 Blood extraction procedure of metformin HCl and sample preparation

Whole blood was collected from rats into heparinised tubes. To separate plasma, tubes were centrifuged at 1000 RCF for 10 min at 4 °C. Plasma (100 μL) was subsequently aliquoted into 1.5 ml Eppendorf® tubes and stored at −80 °C until used. Working standard solutions were prepared by adding an aliquot (20 μL) of metformin HCl solution to 80 μL blank plasma and vortexing for 10 s. For all samples and standard solutions, acetonitrile (500 μL) was then added to the plasma/standard mixture and vortex mixed for 10 min. This was followed by centrifugation at 14,000 RCF for 10 min at 4 °C.The supernatant was then removed from the Eppendorf® tube and transferred to a disposable glass culture tube. The sample extract was then dried under a stream of nitrogen at 35 °C for 40 min using a Zymark TurboVap® LV Evaporator Workstation (McKinley Scientific, Sparta, NJ, USA). The residue was then reconstituted in 100 μL of deionized water. This was vortex mixed for 30 s, collected in an Eppendorf® and centrifuged at 8000 RCF for 5 min at room temperature. The supernatant was then transferred into an Agilent HPLC vial and 10 μL was injected into the HPLC column.

### 2.11 Pharmaceutical analysis

Metformin HCl concentrations were determined using HPLC with UV detection, developed and validated for *in vitro* and *in vivo* samples. *In vitro* samples were centrifuged at 14,000 rpm using an Eppendorf MiniSpin® centrifuge (Eppendorf AG, Hamburg, Germany) for 10 min and diluted by PBS (pH 7.4) prior to HPLC analysis. Metformin HCl concentrations were calculated for *in vitro* samples by comparing the area under the curve (AUC) against external standards, using Eq.(1).

(1) $$\left[\text{Drug}\right]\text{sample}=\frac{\left[\text{Drug}\right]\text{standard}\times \mathrm{AUC}\;\text{sample}}{\mathrm{AUC}\;\text{standard}}\times \text{Dilution factor}$$

Quantification of metformin HCl *in vitro* samples was performed using reversed-phase HPLC. Chromatographic separation was achieved using a Waters Cortecs® C18+ column (150 × 4.6 mm, internal diameter (i.d.), with 2.7 μm packing) (Waters Ireland, LTD, Dublin, Ireland) with isocratic elution and UV detection at 235 nm. The mobile phase was a mixture of acetonitrile: phosphate buffer (containing triethylamine 0.1% and pH adjusted to 3 using phosphoric acid), (30%:70% v/v). Run time was 7 min. The column temperature was 25 °C, the flow rate was 0.3 mL/min and the injection volume was 10 μL.

To account for differences in volume of plasma obtained from each rat, Eq.(2) was used for metformin HCl quantitation of *in vivo* samples.

(2) $$\left[\text{Drug}\right]\text{sample}=\frac{\left[\text{Drug}\right]\text{standard}\times \mathrm{AUC}\;\text{sample}}{\mathrm{AUC}\;\text{standard}}\times \frac{\text{volume}\;\text{of standard}}{\text{volume of sample}}$$

Quantification of metformin HCl in *in vivo* samples was performed using reversed-phase HPLC. Chromatographic separation was achieved using a Waters Cortecs® T3 (150 × 4.6 mm (i.d.) with 2.7 μm packing) analytical column fitted with a guard cartridge of matching chemistry with isocratic elution and UV detection at 235 nm. The mobile phase was a mixture of methanol:phosphate buffer (containing triethylamine 0.1% and pH adjusted to 3 using phosphoric acid), (3%:97% v/v). Run time was 30 min. The column temperature was 25 °C, the flow rate was 0.4 mL/min and the injection volume was 10 μL.

An Agilent 1200® series system (Agilent Technologies UK Ltd., Stockport, UK) was used for all analyses. Agilent ChemStation® Software B.02.01 was used for chromatogram analysis. Correlation analysis along with least squares linear regression analysis was performed on the calibration curves generated, enabling determination of the equations of the line and their coefficients of determination. Limits of detection (LoD) and limits of quantification (LoQ) were determined using a method based on the standard deviation (SD) of the response and the slope of the representative calibration curve, as described in the guidelines from the International Conference on harmonization (ICH) [28].

### 2.12 Statistical analysis

Least squares linear regression analysis, correlation analysis and calculation of SD of line intercepts and residual SDs were all performed using Microsoft® Excel® 2013 (Microsoft Corporation, Redmond, USA). All data were expressed as means ± standard deviation. Where appropriate, the Mann-Whitney-*U* test was performed for comparison of two unpaired groups, when *n* < 5. The Wilcoxon matched-pairs rank test was performed for comparison of two paired groups when n < 5. An unpaired *t*-test was used for comparison of two groups when *n* > 5 and data were normally distributed. The Kruskal-Wallis test with post-hoc Dunn's test was used for comparison of multiple groups. In all cases, *p* < .05 was used to denote statistical significance. Statistical analysis was carried out using GraphPad Prism® version 5.0 (GraphPad Software Inc., San Diego, California).

## 3 Results

### 3.1 Mechanical strength of MNs

The percentage height reduction of MN arrays after applying different compression forces is shown in Fig. 1 (ii). In this mechanical strength test, there was no significant difference in percentage height reduction between the different applied compression forces (*p* > .05). A typical insertion force that would be applied by manual thumb pressure is around 32 N per array [25]. In the present study, the maximum applied compression force was 36.3 N (0.3 N × 121 needles). Even at this force, complete MN array failure was not observed. No fracturing was observed in MN arrays at any of the applied forces. This indicated that MN arrays have sufficient mechanical strength and are unlikely to be broken upon insertion into the skin.

### 3.2 Insertion study using optical coherence tomography

The insertion of MN arrays in Parafilm® M as viewed using OCT is shown in Fig. 1 (iv). The base plate/film surface distance (a), the depth of MN penetration (b) and the pore width (c) were determined following the application of MN arrays to a validated skin model Parafilm® M (Table 2). MN penetration depth was 290.83 μm, the pore width was 157.4 μm and the baseplate/film surface distance was 182.68 μm. This indicated that MN arrays penetrated the Parafilm® M consistently.

**Table 2:** OCT assessment of MNs penetration following application to Parafilm® M *in vitro*.

	Baseplate/film surface distance (μm) (a)	MN penetration depth (μm) (b)	Pore width (μm) (c)
Mean ± SD (*n* = 12)	182.68 ± 27.09	290.83 ± 40.23	157.40 ± 19.49

### 3.3 Characterisation of lyophilised metformin HCl reservoirs

A number of reservoir formulations were prepared using an iterative approach. The favoured formulations were those capable of demonstrating the following characteristics: High metformin HCl drug loading, homogeneous appearance, robust mechanical integrity for ease of handling and rapid dissolution in PBS (pH 7.4). The latter property may suggest that metformin HCl could be readily be released from the reservoir for delivery through the swollen hydrogel network of the MNs following skin application.

All metformin HCl reservoirs were homogenous and robust (Table 3 A and B), except formulae F11 and F12, which contained 55% w/w of metformin HCl and weighed 300 mg and 150 mg, respectively. These formulations were overloaded with metformin HCl and drug crystals were observed on the surface of the reservoirs. According to physical properties and the dissolution time in PBS (pH 7.4), the optimum formulae were formula F6, which contained 75 mg metformin HCl, and F13, which contained 50 mg metformin HCl. F6 and F13 were thus chosen for *in vitro* studies.

**Table 3:** Characteristics of lyophilised metformin HCl reservoirs.

Formula	Thickness (mm)	Dissolution time (min)	Physical properties	Morphology
A.				
F1	3.2	16	Homogeneous, and robust reservoirs	
F2	2.4	12	Homogeneous, and robust reservoirs	
F3	3.7	12	Homogeneous, and robust reservoirs	
F4	2.3	9	Homogeneous, and robust reservoirs	
F5	4.1	8	Homogeneous, and robust reservoirs	
F6	2.5	6	Homogeneous, and robust reservoirs	
B.				
F7	4.5	12	Homogeneous, and robust reservoirs	
F8	2.7	10	Homogeneous, and robust reservoirs	
F9	4.3	17	Homogeneous, and robust reservoirs	
F10	2.8	16	Homogeneous, and robust reservoirs	
F11	5.7	6	Overloaded reservoirs with metformin HCl crystals on surface	
F12	2.3	5	Overloaded reservoirs with metformin HCl crystals on surface	
F13	0.9	3	Homogeneous, and robust reservoirs	

### 3.4 Pharmaceutical analysis of metformin HCl

HPLC methods for the quantification of metformin HCl in either PBS (pH 7.4) or rat plasma, were developed and validated according to ICH guidelines [28]. Calibration curve properties, limits of quantification and detection are presented in Table 4.

**Table 4:** Properties of calibration curves for quantification of metformin HCl in (i) PBS (pH 7.4) *in vitro* and (ii) rat plasma *in vivo*, along with limits of detection and quantification.

Method	Slope	y-Intercept	R^2^	σ	LoD	LoQ
i)*In vitro* (PBS (pH 7.4))	159.36	114.72	0.9994	77.95	1.47 (μg/mL)	4.89 (μg/mL)
ii)*In vivo* (rat plasma)	0.08	12.79	0.9995	3.14	132 ng/mL	402 ng/mL

### 3.5 The effect of lyophilisation on metformin HCl stability

The effect of lyophilisation on the stability of metformin HCl was studied and the percentage recovery values were calculated for both F6 and F13. From formula F6, 93.70% ± 2.68% of metformin HCl was recovered and 100.70% ± 3.90% was recovered from formula F13. There was no significant difference in percentage recovery between formulae F6 and F13 (*p* = .1). This indicated that lyophilisation had no significant influence on the stability of metformin HCl for both formulations F6 and F13.

### 3.6 *In vitro* permeation of metformin HCl

**Fig. 2:**
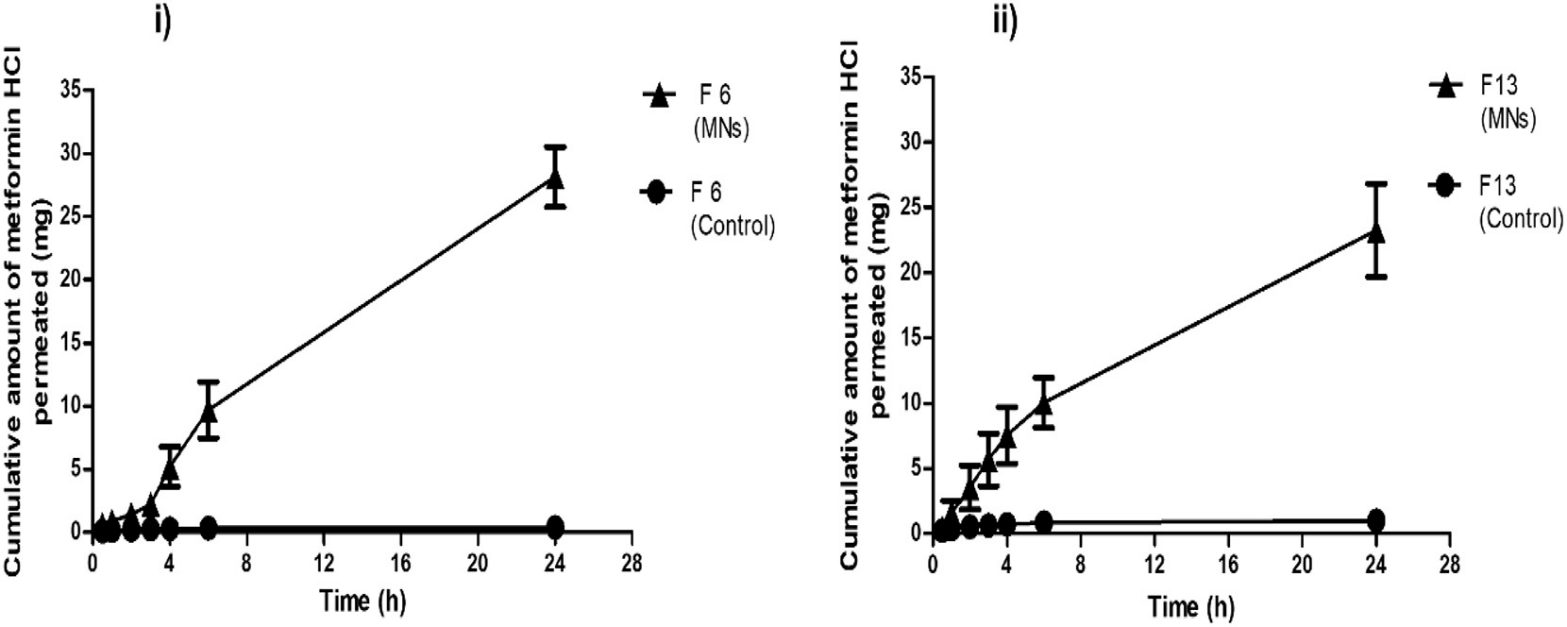
(i) *In vitro* cumulative amount of metformin HCl permeated across dermatomed neonatal porcine skin from reservoir formula F6 (75 mg) in combination with hydrogel-forming MNs in comparison to the control setup with no MNs. (ii) *In vitro* cumulative amount of metformin HCl permeated across dermatomed neonatal porcine skin from reservoir formula F13 (50 mg) in combination with hydrogel-forming MNs in comparison to the control setup with no MNs, (means ± SD, *n* = 6).

Permeation of metformin HCl across dermatomed neonatal porcine skin was significantly enhanced by using MN arrays compared to the control set-up (*p* < .05). Following application of the combined MN with F6, for 6 h and for 24 h, 9.71 ± 2.22 mg and 28.15 ± 2.37 mg metformin HCl were delivered, respectively, in comparison with 0.34 ± 0.39 mg and 0.39 ± 0.39 mg delivered from the control set-up (no MNs) for 6 h and 24 h, respectively (Fig. 2 (i)). In terms of percentage delivery, 12.94 ± 2.96% and 37.53 ± 3.17% of metformin HCl was delivered at 6 h and 24 h, respectively, in comparison to the control set-up, which delivered 0.45 ± 0.52% and 0.52 ± 0.52% at 6 h and 24 h, respectively.

Following application of the combined MN and F13, for 6 h and for 24 h, 10.04 ± 1.92 mg and 23.25 ± 3.58 mg metformin HCl was delivered, respectively, in comparison with 0.85 ± 0.68 mg and 1.01 ± 0.84 mg delivered from the control set-up (no MNs), for 6 h and 24 h, respectively (Fig. 2 (ii)). In terms of percentage delivery, at 6 h and 24 h, 20.07 ± 3.84% and 46.50 ± 7.16% of metformin HCl was delivered, respectively, in comparison to the control set-up, which delivered 1.71 ± 1.36% and 2.01 ± 1.68%, at 6 h and 24 h, respectively.

**Fig. 3:**
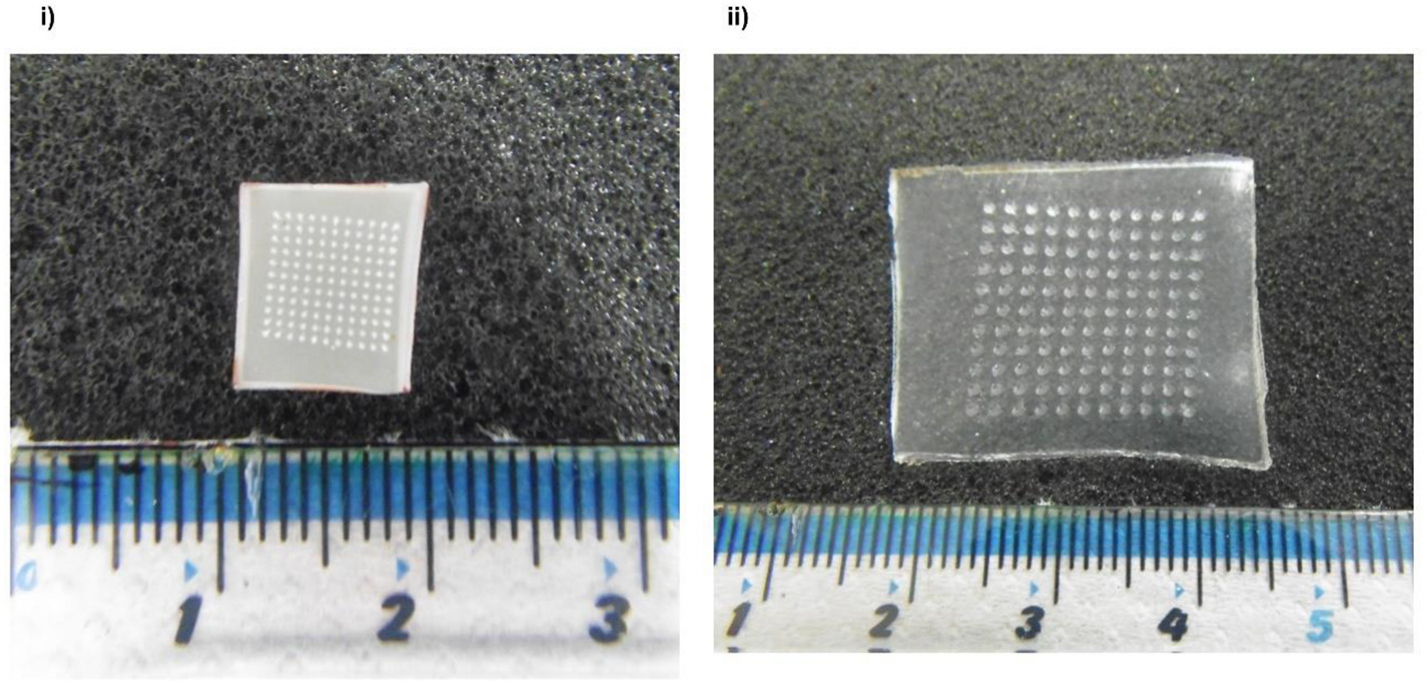
Digital images of (i) MN array before swelling in PBS (pH 7.4). (ii) MN array after swelling in PBS (pH 7.4) for 24 h.

Fig. 3 (i), (ii) shows digital images of a MN array before swelling in PBS (pH 7.4) and after swelling in PBS (pH 7.4) for 24 h, respectively.This indicates the now well-known-capacity of such systems to swell [1].

Formula F13 was chosen for the *in vivo* study, as it had faster dissolution (3 min) compared to F6, which had a dissolution time of 6 min. Rapid dissolution of a drug reservoir in PBS (pH 7.4) indicates that metformin HCl could readily be released from the reservoir and thus be available for permeation through a swollen hydrogel network. In addition, F13 had a lower thickness (0.90 mm) compared to F6 (2.47 mm). Reservoir thickness is an important aspect. In previous *in vivo* work within our Group, it was found that MN patches need to be relatively thin to ensure that rats will tolerate them for 24 h. If the patch is too bulky and cumbersome, it can interfere with the rat's natural behaviour and they will strive to remove it over the course of the study period. Furthermore, when considering this delivery system as a final product in humans, it is likely that patients would prefer patches that do not extend significantly outwards from the surface of the skin.

### 3.7 *In vivo* study

**Fig. 4:**
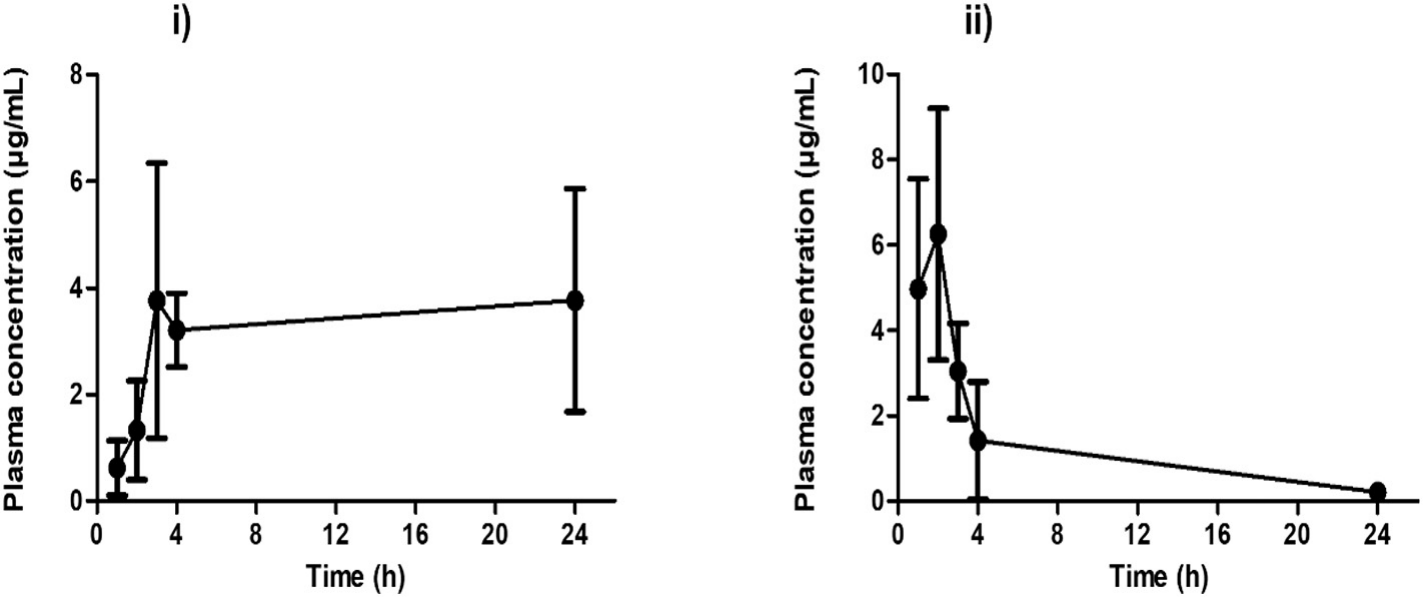
(i) The *in vivo* plasma profiles of metformin HCl following application of hydrogel-forming MNs and solid metformin HCl-containing reservoir (F13) integrated patches at a dose of 100 mg. (ii) The *in vivo* plasma profiles of metformin HCl following oral gavage at a dose of 100 mg/kg of rat weight, (means ± SD, *n* at least = 4, for 24 h, *n* = 8).

Each rat in the MNs transdermal treatment group had two integrated MN patches applied to their back. As can be seen from the plasma profile (Fig. 4 (i)), metformin HCl was detected in rat plasma at 1 h post MN application at a concentration of 0.62 ± 0.51 μg/mL. The concentration increased to 3.76 ± 2.58 μg/ml at 3 h and then decreased slightly at 4 h to 3.21 ± 0.69 μg/mL. However, a maximal concentration of 3.77 ± 2.09 μg/ml was achieved for the 100 mg dose, the total of two applied 50 mg MN patches, at 24 h.

Each rat in the oral control group received an oral solution of metformin HCl 100 mg/kg. As can be seen from the plasma profile (Fig. 4 (ii)), metformin HCl was detected in the rat plasma at 1 h post-oral gavage administration at a concentration of 4.97 ± 2.57 μg/mL. The concentration increased to a maximum of 6.25 ± 2.94 μg/m, at 2 h. At 4 h, metformin HCl concentration decreased to 1.42 ± 1.37 μg/ml and continued to decrease until the 24 h endpoint.

**Fig. 5:**
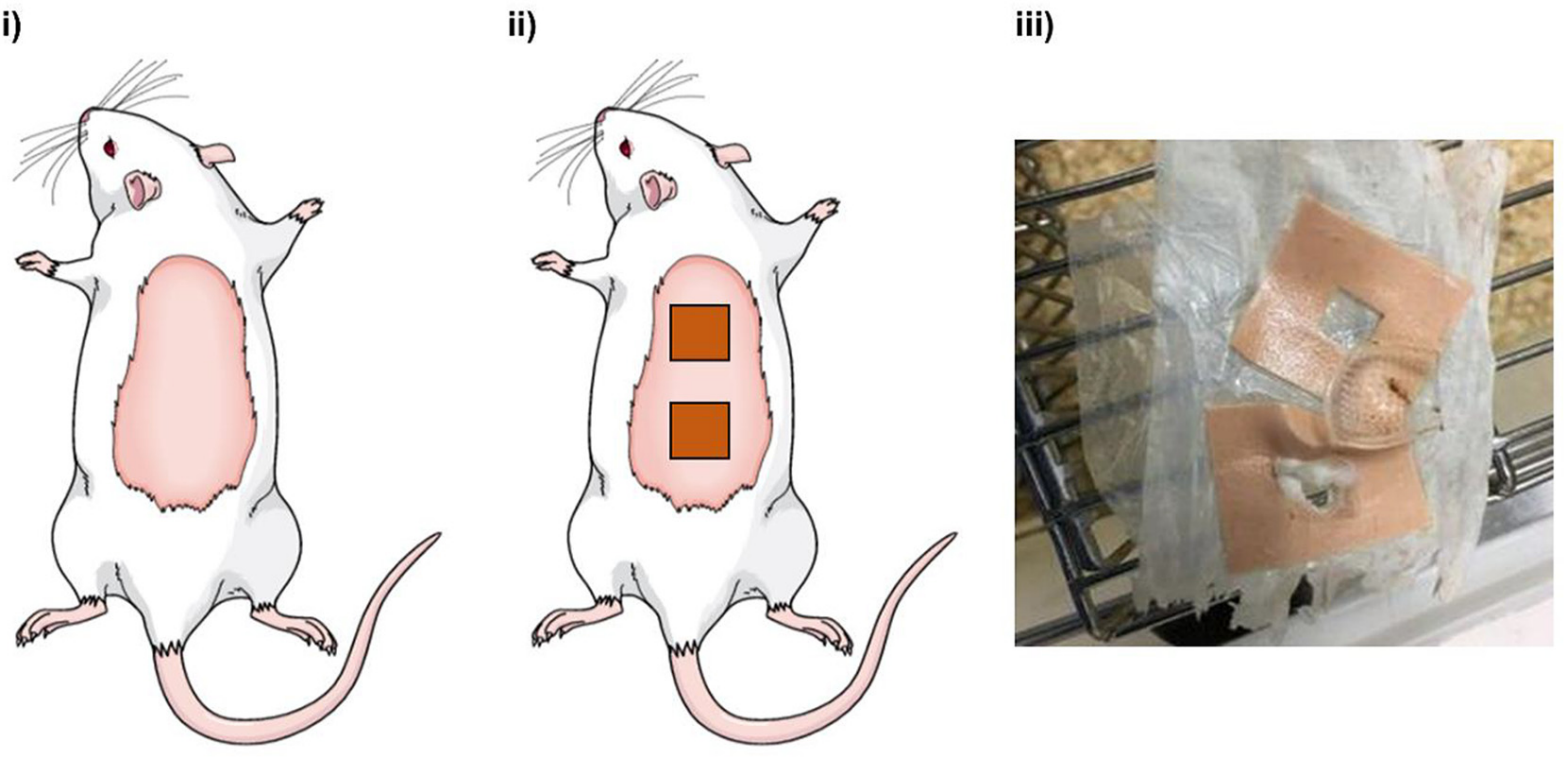
Representative images of *in vivo* study displaying MNs application process. (i) Shaving the rat hair on the back, (ii) MN arrays with drug reservoir patches applied *in situ*, (iii) MN arrays removed from the back of a rat after 24 h with the adhesive backing removed and the swollen MN arrays.

Fig. 5 shows the *in vivo* MN application process. Fig. 5 (iii) shows MN arrays that have swelled extensively, with each drug reservoir having dissolved fully at 24 h. MN arrays were removed intact from the skin, leaving no polymeric residues behind. No oedema was observed at the site of application. Following removal of patches, some rats displayed only minimal erythema, which resolved fully within 1 h post MN patch removal.

Pharmacokinetic parameters are presented in Table 5. C_ss_ (steady-state plasma concentration) was calculated using Eq.(3), where *AUC* is the area under the plasma concentration/time curve and *t* is time.

(3) $$C_{\mathrm{ss}}=\frac{{\mathrm{AUC}}_{t_{0-24}}}{t}$$

**Table 5:** Pharmacokinetic parameters of metformin HCl in the MNs transdermal treatment group and in the oral control group (means ± SD, n at least = 4, for 24 h, *n* = 8).

Parameter	MNs (dose = 50 mg/MN patch)	Oral (dose = 100 mg/kg)
AUC (μg·h/mL)	77	31.29
T_max_ (h)	24	2
C_max_ (μg/mL)	3.77 ± 2.09	6.25 ± 2.94
C_ss_ (μg/mL)	3.2	Cannot be calculated for single oral dose

T_max_ (time of maximum concentration observed) was 24 h in the MN transdermal-treated rat group and C_ss_ was 3.2 μg/mL.

Relative transdermal bioavailability of metformin HCl using hydrogel-forming MN was determined according to Eqs.(4), (5) by dividing AUC of the plasma profile of metformin HCl in the MNs transdermal treatment group by AUC of the plasma profile of metformin HCl in the oral control group. Where ***AUC***_***t*0**−**24**_ is the area under the curve from zero time to 24 h, F is the bioavailability, X_0_ is the initial dose, Cl is the clearance of metformin HCl.

(4) $${\mathrm{AUC}}_{t0-24}=\frac{FX_{0}}{\mathrm{Cl}}$$

(5) $$\frac{{\mathrm{AUC}}_{t0-24\;}\left(\mathrm{MNs}\right)}{{\mathrm{AUC}}_{t0-24}\;\left(\mathrm{Oral}\right)}=\frac{FX_{0\;\left(\mathrm{MNs}\right)}}{FX_{0\;\left(\mathrm{oral}\right)}}$$

In the MNs transdermal treatment group, X_0_ was 100 mg (two MN patches), X_0_ in the oral control group was 22.4 mg, (at an oral dose of 100 mg/kg, the average weight of rats in the oral gavage group was 224.5 g ± 16.57 g). Therefore, the relative transdermal bioavailability of metformin HCl using hydrogel-forming MNs is [F(MNs)/F(oral)] and it was calculated as 0.6 of the oral bioavailability (F(oral)). The oral bioavailability of metformin HCl (F(oral)) is known to be approximately 50%, or 0.5 [23]. F(MNs) is the transdermal bioavailability of metformin HCl when delivered using our MNs and it was calculated as 0.3 (30%). This means that approximately 30 mg of metformin HCl was delivered transdermally using the MN patches during the study.

To achieve equivalent delivery of metformin HCl from a 500 mg oral tablet, a hydrogel-forming MN patch containing 833 mg would be required. As these MN systems contain 50 mg/MN patch and has an array area of 0.49 cm^2^, this means that the expected patch size that the patient may need to apply to their skin is around 8 cm^2^ (containing 833 mg metformin HCl) every 24 h.

## 4 Discussion

Presented in this study is a novel combination of a metformin HCl-containing lyophilised drug reservoir and a hydrogel-forming MN array and aims to enhance the transdermal delivery of this antidiabetic drug. Hydrogel-forming MNs have demonstrated the ability to enhance the transdermal delivery of many therapeutic substances with a wide range of physicochemical properties [1,3,4]. This highlights the versatility of this MN type and shows that hydrogel-forming MNs have considerable promise for commercial success. When inserted into the skin, hydrogel-forming MNs are in contact with interstitial fluid. MNs swell and create porous aqueous microconduits through which drug substances can diffuse and reach the dermal microcirculation. The aqueous network created by these MNs is suitable for transdermal delivery of drugs with a relatively high degree of water solubility, for example, metformin HCl.

The primary objectives of this work included formulating a well-formed lyophilised drug reservoir using biocompatible materials with a high drug loading of metformin HCl, rapidly dissolving in aqueous fluid and yielding high drug recovery. The ingredients selected for the lyophilised drug reservoir preparation have been previously used and shown to have the required properties [3]. To characterise the drug reservoirs, physical tests were conducted to assess the suitability of the formulation for use in the transdermal application. Among the prepared drug reservoirs formulations, two were selected (F6, F13) based on formulation integrity, high metformin HCl content and rapid dissolution rates in PBS (pH 7.4).MN swelling, followed by fluid movement through the swollen microstructures, is the key factor in facilitating drug reservoir dissolution and in creating the micropores though which drug diffusion occurs. The swelling behaviour of hydrogel-forming MNs in PBS (pH 7.4) has been evaluated previously by our Group [3]. In addition, MNs should have sufficient mechanical strength and demonstrate consistent insertion properties to achieve successful application of MN array into the dermatomed porcine skin *in vitro* and rat skin *in vivo*.

MN inserted efficiently into the validated skin model Parafilm® M [25] [[1], [2], [3],^24^,^25^]. Hydrogel-forming MNs also showed sufficient mechanical strength to penetrate skin *in vitro* and *in vivo*, consistently.

The *in vitro* permeation studies carried out with formulae F6 and F13 with hydrogel-forming MN arrays across neonatal porcine skin *via* the Franz diffusion cell technique demonstrated that for F6, 12.9 ± 2.96% (9.71 mg) and approximately 37.5 ± 3.17% (28.15 mg) of metformin HCl loadings were successfully delivered at 6 h and 24 h, respectively. For F13, 20.07 ± 3.84% (10.04 mg) of metformin HCl was delivered at 6 h and approximately 46.50 ± 7.16% (23.25 mg) of the metformin HCl was successfully delivered at 24 h.

Following insertion, the MNs imbibe fluid, with subsequent swelling and expansion of the hydrogel matrix, this facilitated the diffusion of metformin HCl drug molecules through the micropores created in the skin. The imbibed fluid causes dissolution of the lyophilised drug reservoir. With time, MN swelling increased and caused higher amounts of metformin HCl to permeate through the MN microchannels. Upon visual inspection of the Franz cell donor compartment post-experiment, it was obvious that there was complete dissolution of the drug reservoir.

A safe oral dose of metformin HCl for rats is 100–200 mg/kg [26]. Taking into consideration the mean weight of a rat (250 g) and the mean oral bioavailability of metformin HCl (50–60%) [25], it was estimated that, for a dose of 100–200 mg/kg, 15–30 mg would reach the systemic circulation. To ensure a safe oral dose was employed, 100 mg/kg was selected, thus minimizing the chance of toxicity and side effects.

Following the *in vitro* permeation studies, it was clear that 46.50 ± 7.16%, (23.25 ± 3.58 mg) of metformin HCl in formulation F13 was released into the donor compartment. Using this as a guide for planning *in vivo* transdermal dosing, assuming 25–30% of metformin HCl will be successfully delivered to the rats over 24 h, MN patches could, therefore, include 100 mg metformin HCl.

The results obtained in the *in vivo* experiment suggest that the use of hydrogel-forming MN arrays offer the potential for successful transdermal delivery of metformin HCl. It is important to highlight that metformin HCl plasma concentration was shown to be sustained over 24 h. This may indicate that the drug continues to be released from the MN patch while it was being cleared from the body of the rats.

Once the drug has diffused through the MNs, uptake by the dermal microcirculation is likely to be rapid. By the end of the experiment runtime, it was noted that an almost constant plasma concentration of metformin HCl was maintained. Metformin HCl delivered from an integrated MN patch yielded plasma concentrations in the rat model, reaching the target therapeutic concentration in humans of (1–5) μg/ml [29,30].

The relative transdermal bioavailability of metformin HCl when using hydrogel-forming MNs was estimated as 0.6 of its oral bioavailability. The transdermal bioavailability of metformin HCl when using hydrogel-forming MNs was estimated as 0.3. This means that 30% of the drug loading would be delivered within 24 h.It is recognized that the dose of metformin HCl used in this study is much lower than the oral human dose. In the plasma profile of metformin HCl, it is obvious that the steady-state concentration was achieved within the duration of this experiment (24 h). In transdermal delivery, the rate of absorption is slower than oral delivery, due to the lag time associated with initial swelling of MN patches.Actually, it is important to consider that the pharmacokinetics of a rat and a human are quite different, but the results of this small-scale study are promising. When cautiously extrapolated, a human metformin HCl dose of 500 mg could be achieved with a patch size of approximately 8 cm^2^, containing a drug load of 833 mg metformin HCl.

Potential approaches to improve the bioavailability of transdermally-delivered metformin HCl in future may include changing the type of crosslinker. Hydrogel-forming MNs using a higher molecular weight crosslinker could increase the swelling of MNs. Further changes to the type of drug reservoir may also further enhance delivery of metformin HCl. Hydrogel-forming MNs are clearly a promising technology that could be used to enhance transdermal delivery of a range of therapeutic substances [31]. The aim of this study was to establish preliminary data to investigate the potential for transdermal delivery of metformin HCl by hydrogel-forming MNs. We have provided ‘proof of concept’ evidence that metformin HCl can be delivered transdermally using hydrogel-forming MNs.

## 5 Conclusion

The work presented here reports successful design and evaluation of a combined hydrogel-forming MN array / drug reservoir transdermal patch. In *in vivo* experiments, therapeutic doses of metformin HCl were delivered to rats in a sustained manner, highlighting the potential of this delivery route for metformin HCl administration. This type of system may provide an alternative mode of delivery for patients and help to minimize gastrointestinal side effects and small intestine absorption variations associated with conventional oral delivery. In pathway to commercialization of this technology, especially when manufactured on a large scale, cost will be an important issue and will need to be minimized, especially for generic, off-patent drugs, such as metformin, where profit margins are tight. Hydrogel-forming MNs represent a promising technology that could be used for transdermal delivery of many other established drugs with high oral doses. If clinical benefits can be shown, then perhaps healthcare providers would be willing to pay slightly more than they currently do for oral medicines.
